# Combination of the Fibrosis 4 Index and Carbohydrate Antigen 125 to Predict Morbidity and Mortality in Acute Heart Failure

**DOI:** 10.31083/RCM42797

**Published:** 2025-12-22

**Authors:** Franco Appiani, Raquel López-Vilella, Víctor Donoso, Julia Martínez-Solé, Valero Soriano, Sara Huélamo, Susana Beltrán, Ana Elisa Astudillo, Mireia Company, Borja Guerrero, Luis Martínez, Luis Almenar-Bonet

**Affiliations:** ^1^Heart Failure and Transplant Unit, Hospital Universitari i Politècnic La Fe, 46007 Valencia, Spain; ^2^Cardiology Department, Hospital Universitari i Politècnic La Fe, 46007 Valencia, Spain; ^3^Centro de Investigación Biomédica en Red de Enfermedades Cardiovasculares (CIBERCV), Instituto de Salud Carlos III, 28029 Madrid, Spain

**Keywords:** FIB-4, CA125, acute heart failure, biomarkers, prognosis, worsening, mortality

## Abstract

**Background:**

The implementation of the fibrosis 4 (FIB-4) index was initially associated with hepatic dysfunction; however, this index may also provide prognostic information in heart failure (HF). Thus, this study aimed to assess whether combining the FIB-4 and carbohydrate antigen 125 (CA125) indices in patients hospitalized for acute heart failure (AHF) can identify subgroups with differing risks of morbidity and mortality.

**Methods:**

This retrospective study included 402 patients consecutively admitted for AHF between January 2023 and December 2024, after excluding elective admissions (n = 403), inter-hospital transfers (n = 232), and low-output cases (n = 51). Patients were stratified into four groups according to the FIB-4 score (<1.3 or high) and CA125 value (≤50 U/mL or high): Group 1 (low FIB-4 + low CA125; n = 43), Group 2 (low FIB-4 + high CA125; n = 57), Group 3 (high FIB-4 + low CA125; n = 117), and Group 4 (high FIB-4 + high CA125; n = 185). Clinical, echocardiographic, therapeutic, and laboratory variables were analyzed, as well as morbidity (HF-related emergency visits and readmissions) and all-cause mortality.

**Results:**

Patients with both elevated FIB-4 and CA125 values had a higher prevalence of systemic/mixed congestion (*p* < 0.01), higher N-terminal pro-B-type natriuretic peptide (NT-proBNP) levels (*p* < 0.01), and less frequent inspiratory inferior vena cava (IVC) collapse (*p* < 0.01). Although no survival differences were observed (*p* = 0.29), morbidity was significantly higher in group 4: more worsening episodes per patient (*p* = 0.0001), increased HF readmissions (*p* = 0.004), and more emergency visits (*p* = 0.001). The FIB-4 index correlated positively with worsening episodes (*p* < 0.0001), and the CA125 value showed a trend with mortality. No significant correlation was found between FIB-4 and CA125 or between FIB-4 and mortality (*p* > 0.1).

**Conclusions:**

The FIB-4 index may be a useful indicator in AHF. Elevated values at admission for decompensation, in combination with high CA125 levels, can be used to identify a subgroup of patients with poor short- to medium-term outcomes, particularly in terms of worsening. Further studies are needed to determine the actual utility of the FIB-4 index in the context of AHF.

## 1. Introduction

Acute heart failure (AHF) is a condition associated with high morbidity and mortality and represents one of the leading causes of hospital admission in cardiology departments [1]. Systemic congestion, particularly hepatic congestion, plays a key role not only in the clinical presentation but also in preventing hospitalizations and disease progression [2]. Proper assessment of the congestion status is essential for therapeutic management and risk stratification in these patients [3].

The FIB-4 index, originally developed to estimate the degree of liver fibrosis in patients with viral hepatitis, has emerged as a prognostic tool in heart failure (HF) [4, 5]. This index, calculated from age, aspartate aminotransferase (AST), alanine aminotransferase (ALT), and platelet count, may reflect the impact of hepatic congestion in both chronic and acute HF scenarios. Recent studies have shown that elevated FIB-4 values are associated with higher mortality and rehospitalization in AHF patients, suggesting that congestion-induced liver dysfunction is an indirect marker of the severity of the congestive syndrome [6, 7].

On the other hand, carbohydrate antigen 125 (CA125), widely used in oncology as a marker for ovarian neoplasms, has shown utility as a biomarker of systemic volume overload in HF [8]. CA125 levels are known to rise in response to systemic inflammation and endothelial activation under hypervolemic conditions. Several studies have documented that elevated CA125 concentrations in HF patients correlate with greater clinical severity, poorer response to diuretic therapy, and increased risk of adverse events, including hospitalization and death [8, 9, 10, 11].

These distinct biological pathways suggest that each biomarker could provide complementary information and might enhance risk stratification in AHF. The primary objective of the study was to analyze the clinical characteristics associated with the combined blood levels of these biomarkers, compare short- and mid-term survival, determine their relationship with morbidity and mortality, and assess the correlation between both serum markers.

## 2. Materials and Methods

This was a retrospective study based on a database of patients consecutively admitted for AHF at the Department of Cardiology of a tertiary care hospital. Data collection was performed during hospitalization and was extracted and stored by a team of clinical cardiologists specialized in HF. Recruitment was conducted consecutively over 2 years (January 2023–December 2024), and 1088 patients were initially considered. Exclusion criteria included elective admissions (n = 403), inter-hospital transfers (n = 232), and low-output clinical syndromes (n = 51). The final sample comprised 402 patients.

**Fig. 1.**
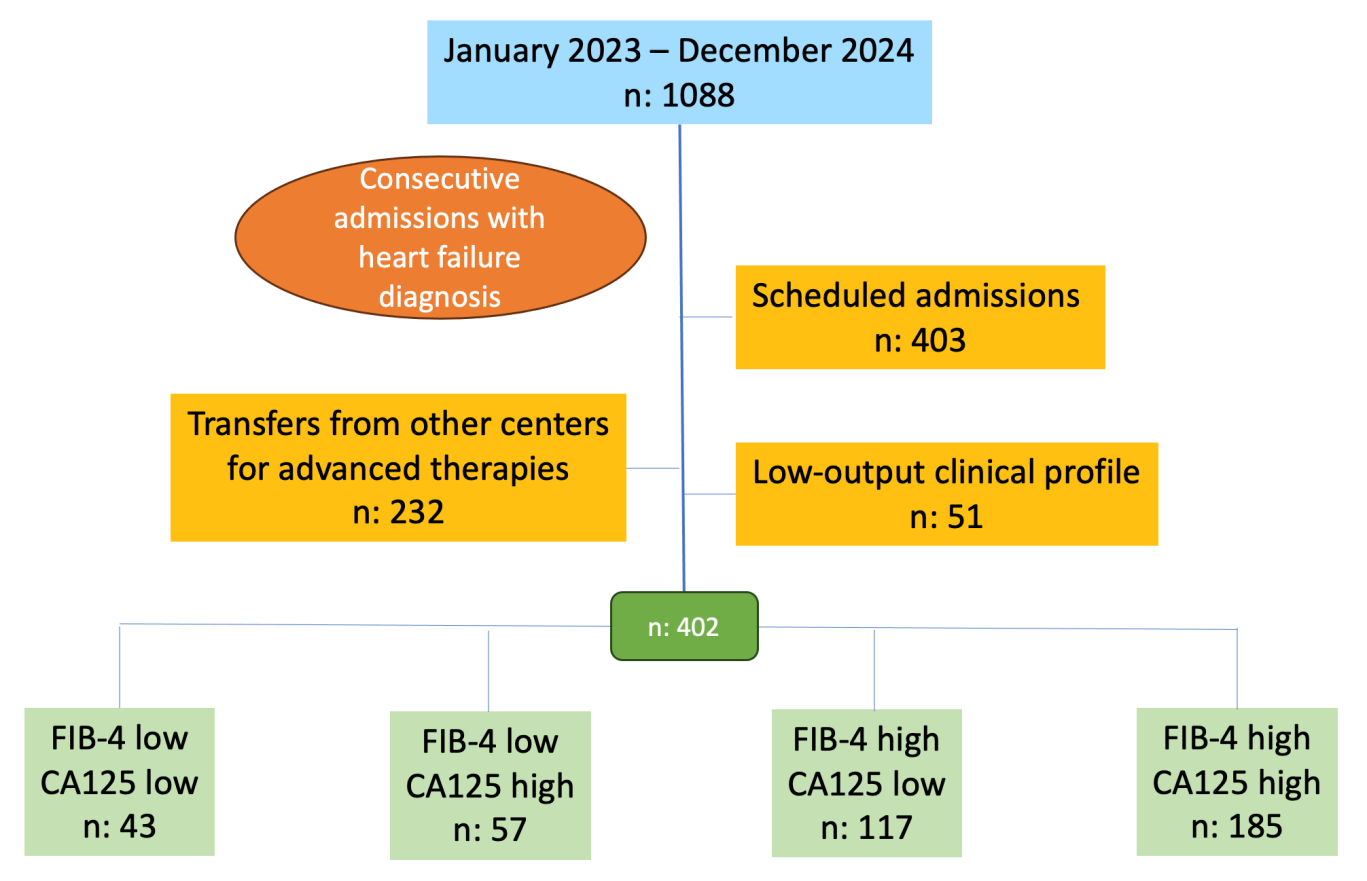
**Study flow chart**. Groups were defined according to biomarker thresholds: FlB-4 low: FlB-4 <1.3; FlB-4 high: FlB-4 ≥1.3; CA125 low: CA125 ≤50 U/mL; CA125 high: CA125 >50 U/mL. Abbreviation: CA125, Carbohydrate Antigen 125.

FIB-4 was calculated using the formula: FIB-4 Index = [Age (years) × AST (U/L)] / [Platelet count (10^9^/L) × √ALT (U/L)] [4]. Four groups were formed based on biomarker combination, with low FIB-4 defined as <1.3 and normal CA125 as ≤50 U/mL. The resulting groups were: Group 1: Low FIB-4 + Low CA125 (n = 43); Group 2: Low FIB-4 + High CA125 (n = 57); Group 3: High FIB-4 + Low CA125 (n = 117); Group 4: High FIB-4 + High CA125 (n = 185) (Fig. 1). Variables of interest included: Clinical: baseline characteristics, comorbidities and clinical profile; Echocardiographic: functional evaluation of both ventricles and inferior vena cava (IVC); Therapeutic: medications at the time of admission; Laboratory: standard parameters assessed with a specific panel for decompensated HF [12, 13].

Groups were compared, and follow-up analysis was performed for overall survival (all-cause mortality), morbidity (worsening—HF-related emergency visits and/or HF rehospitalizations), and correlation between both biomarkers. The study was conducted in accordance with the Declaration of Helsinki. The research protocol was approved by the Biomedical Research Ethics Committee of Hospital Universitari i Politècnic La Fe (reference code COMBICAR).

### Statistical Analysis

Survival analyses were performed using a time-to-first-event approach. For other outcomes such as hospitalizations, emergency visits, and worsening HF episodes, the total number of events per patient was calculated and summarized descriptively, without modeling recurrent events.

Categorical variables were expressed as percentages and continuous variables as mean (standard deviation) or median (interquartile range), depending on whether they followed a normal distribution, which was assessed using the Kolmogorov-Smirnov test. Comparisons between groups were performed using the Chi-square test with Yates’ correction when applicable for qualitative variables, and Student’s *t*-test or Mann-Whitney U test for continuous variables depending on distribution.

There were no missing data for the primary outcomes (mortality and worsening HF) or for the main biomarkers (FIB-4 and CA125). For the remaining variables, missing data were <5% and therefore no imputation procedures were required.

Survival was analyzed using Kaplan-Meier curves, with comparisons made using the Log-Rank test. The Pearson correlation test was used to analyze relationships between the variables of interest. A *p*-value < 0.05 was considered statistically significant. Statistical analyses were performed using IBM SPSS Statistics v27® (IBM Corp, Armonk, NY, USA) and Stata® Stata Statistics 16.1 (StataCorp LLC, College Station, TX, USA). Figures were generated using SPSS and PowerPoint v16.1 softwares.

## 3. Results

### 3.1 Clinical Profile and Baseline Characteristics

Most patients were male (62%) with a mean age of 72 ± 12 years. There was a high prevalence of comorbidities: hypertension (78%), dyslipidemia (61%), and diabetes mellitus (49%). The main etiology of HF was valvular (32%), followed by ischemic origin (25%). A significant percentage of patients presented with atrial fibrillation (59%). Age was higher in subgroups with elevated FIB-4 (*p* < 0.01). Overall analysis showed few significant differences between groups (Table 1). New York Heart Association (NYHA) functional class prior to admission was class II in 50% and class III or IV in 35%. The most common clinical profile was pulmonary congestion. A total of 34% had at least one hospital admission in the previous year. Although few relevant clinical differences were observed across groups, mixed congestion patterns were more common in the group with both biomarkers elevated (Table 2). Among patients with both markers in the normal range, 27.9% exhibited systemic congestion patterns (mixed or isolated); this proportion increased to 38.6% among those with elevated CA125 alone. Adding FIB-4 ≥1.3 significantly increased the frequency to 59.5% (OR: 2.33, 95% CI: 1.27–4.29).

**Table 1.** **Baseline characteristics**.

		FIB-4 <1.3	FIB-4 <1.3	FIB-4 ≥1.3	FIB-4 ≥1.3	*p*	All patients
		CA125 ≤50 U/mL	CA125 >50 U/mL	CA125 ≤50 U/mL	CA125 >50 U/mL		n: 402
		n: 43	n: 57	n: 117	n: 185		
Age (years)#		67 ± 15	64 ± 15	75 ± 10	76 ± 10	<0.001	72.4 ± 12.1
Sex (male)%		23 (53.5%)	41 (71.9%)	70 (59.8%)	115 (62.2%)	0.267	249 (61.9%)
Baseline heart disease						0.143	
	HT	8 (18.6%)	4 (7.0%)	13 (11.1%)	16 (8.6%)		41 (10.2%)
	Ischaemic	10 (23.3%)	15 (26.3%)	31 (26.5%)	45 (24.3%)		101 (25.1%)
	IDCM	2 (4.6%)	6 (10.5%)	16 (13.7%)	27 (14.6%)		51 (12.7%)
	Valvular	17 (39.5%)	14 (24.6%)	41 (35.0%)	55 (29.7%)		127 (31.6%)
	Other	6 (14.0%)	18 (31.6%)	16 (13.7%)	42 (22.7%)		82 (20.4%)
Previous CVS		11 (25.6%)	13 (22.8%)	28 (23.9%)	49 (26.5%)	0.640	101 (25.1%)
HT		35 (81.4%)	43 (75.4%)	95 (81.2%)	141 (76.2%)	0.112	314 (78.1%)
Dyslipidaemia		24 (55.8%)	39 (68.4%)	74 (63.2%)	110 (59.5%)	0.111	247 (61.4%)
Diabetes mellitus		20 (46.5%)	32 (56.1%)	48 (41.0%)	95 (51.4%)	0.170	195 (48.5%)
Active smoking*		10 (23.3%)	23 (40.4%)	40 (34.2%)	80 (43.2%)	0.128	153 (38.1%)
Active drinking		1 (2.3%)	6 (10.5%)	9 (7.7%)	16 (8.6%)	0.177	32 (8.0%)
COPD		6 (14.0%)	7 (12.3%)	13 (11.1%)	34 (18.4%)	0.067	60 (14.9%)
SAHS		3 (7.0%)	8 (14.0%)	20 (17.1%)	19 (10.3%)	0.533	50 (12.4%)
Obesity		8 (18.6%)	10 (17.5%)	19 (16.2%)	23 (12.4%)	0.113	60 (14.9%)
Renal failure		16 (37.2%)	24 (42.1%)	49 (41.9%)	72 (38.9%)	0.185	161 (40.0%)
Hypothyroidism		7 (16.3%)	6 (10.5%)	10 (8.5%)	20 (10.8%)	0.418	43 (10.7%)
Atrial fibrillation		24 (55.8%)	38 (66.7%)	63 (53.8%)	110 (59.5%)	0.364	235 (58.5%)
Stroke		5 (11.6%)	9 (15.8%)	10 (8.5%)	14 (7.6%)	0.414	38 (9.5%)
PVD		4 (9.3%)	2 (3.5%)	15 (12.8%)	15 (8.1%)	0.191	36 (9.0%)
Peritoneal dialysis		1 (2.3%)	0 (0.0%)	0 (0.0%)	2 (1.1%)	0.507	3 (0.7%)

# Kolmogorov-Smirnov <0.05. Median and interquartile range. Values are expressed as absolute numbers and percentage (in parentheses). * Active smoker or ex-smoker <1 year. Abbreviations: CA125, Carbohydrate Antigen 125; COPD, chronic obstructive pulmonary disease; CVS, cardiovascular surgery; HT, hypertension; IDCM, idiopathic dilated cardiomyopathy; PVD, peripheral vascular disease; SAHS, sleep apnea-hypopnea syndrome.

**Table 2.** **Clinical profile of patients**.

		FIB-4 <1.3	FIB-4 <1.3	FIB-4 ≥1.3	FIB-4 ≥1.3	*p*	All patients
		CA125 ≤50 U/mL	CA125 >50 U/mL	CA125 ≤50 U/mL	CA125 >50 U/mL		n: 402
		n: 43	n: 57	n: 117	n: 185		
*De novo* HF		12 (27.9%)	19 (33.3%)	35 (29.9%)	45 (24.3%)	0.474	111 (27.6%)
Functional class (NYHA)						0.239	
	I	7 (16.3%)	11 (19.3%)	20 (17.1%)	22 (11.9%)		60 (14.9%)
	II	19 (44.2%)	30 (52.6%)	64 (54.7%)	87 (47.0%)		200 (49.8%)
	III, IV	17 (39.5%)	16 (28.1%)	33 (28.2%)	76 (41.1%)		142 (35.3%)
Congestive pattern						<0.01	
	Pulmonary	31 (72.1%)	35 (61.4%)	85 (72.6%)	75 (40.5%)		226 (56.2%)
	Systemic	2 (4.7%)	4 (7.0%)	8 (6.8%)	30 (16.2%)		44 (10.9%)
	Mixed	10 (23.2%)	18 (31.6%)	24 (20.5%)	80 (43.2%)		132 (32.8%)
Cause of decompensation						0.028	
	Progression	7 (16.2%)	21 (36.8%)	28 (23.9%)	59 (31.9%)		115 (28.6%)
	Infections	5 (11.6%)	6 (10.5%)	22 (18.8%)	22 (11.9%)		55 (13.7%)
	Arrhythmias	12 (27.9%)	13 (22.8%)	22 (18.8%)	39 (21.1%)		86 (21.4%)
	Unknown	3 (7.0%)	12 (21.1%)	16 (13.7%)	27 (14.6%)		58 (14.4%)
	Other	16 (37.2%)	5 (8.8%)	29 (24.8%)	38 (20.5%)		88 (21.9%)
≥1 Admission in the previous year		15 (34.9%)	22 (38.6%)	35 (29.9%)	65 (35.1%)	0.319	137 (34.1%)
Inhospital days#		8 [5–10]	6 [5–9]	7 [4–10]	8 [5–13]	0.0782	8 [5–11]
SBP (mmHg)#		132.1 ± 32.2	141.2 ± 22.9	136.3 ± 31.1	134.7 ± 49.2	0.332	134 ± 39
DBP (mmHg)#		74.9 ± 18.8	77.1 ± 14.0	75.2 ± 16.2	77.8 ± 13.9	0.435	77.6 ± 15
HR (bpm)#		89.3 ± 19.7	90.6 ± 28.1	84.6 ± 27.6	86.9 ± 23.5	0.463	87 ± 25

# Kolmogorov-Smirnov <0.05. Median and interquartile range. ANOVA de Kruskal-Wallis. Values are expressed as absolute numbers and percentage (in parentheses). Abbreviations: CA125, Carbohydrate Antigen 125; SBP, Systolic Blood Pressure; DBP, Diastolic Blood Pressure; HR, Heart Rate; NYHA, New York Heart Association.

### 3.2 Treatment and Laboratory Results at Admission

A high proportion of patients were receiving renin-angiotensin system inhibitors (angiotensin-converting enzyme (ACE) inhibitors, angiotensin receptor blockers (ARBs), or angiotensin receptor and neprilysin inhibitor (ARNI)) (52%), beta-blockers (62%), and loop diuretics (65%) at admission. Mineralocorticoid receptor antagonists (MRAs) were used in 31% of patients and sodium-glucose cotransporter-2 inhibitors (SGLT2i) in 42%. Comparative analysis between groups did not show statistically significant differences (*p* > 0.05) (Table 3). Most laboratory parameters at admission were within normal ranges. Estimated glomerular filtration rate (eGFR) was around 50 mL/min/1.73 m^2^, and N-terminal pro-B-type natriuretic peptide (NT-proBNP) levels exceeded 6000 pg/mL, with significant differences between groups. As expected, age, liver function tests, and platelet count, which are components of the FIB-4 formula, differed significantly between high and low FIB-4 groups (Table 4).

**Table 3.** **Treatment prior to admission**.

	FIB-4 <1.3	FIB-4 <1.3	FIB-4 ≥1.3	FIB-4 ≥1.3	*p*	All patients
	CA125 ≤50 U/mL	CA125 >50 U/mL	CA125 ≤50 U/mL	CA125 >50 U/mL		n: 402
	n: 43	n: 57	n: 117	n: 185		
ACEI/ARB/ARNI	24 (55.8%)	33 (57.9%)	61 (52.1%)	89 (48.1%)	0.546	207 (51.5%)
Beta-blockers	25 (58.1%)	35 (61.4%)	74 (63.2%)	115 (62.2%)	0.948	249 (61.9%)
MRA	11 (25.6%)	16 (28.1%)	35 (29.9%)	62 (33.5%)	0.698	124 (30.8%)
SGLT2i	15 (34.9%)	27 (47.4%)	44 (37.6%)	84 (45.4%)	0.339	170 (42.3%)
Loop diuretic	28 (65.1%)	35 (61.4%)	70 (59.8%)	127 (68.6%)	0.431	260 (64.7%)
Thiazides	11 (25.6%)	13 (22.8%)	21 (17.9%)	36 (19.5%)	0.694	81 (20.1%)
Tolvaptan	0 (0.0%)	2 (3.5%)	2 (1.7%)	3 (1.6%)	0.610	7 (1.7%)
Acetazolamide	1 (2.3%)	2 (3.5%)	3 (2.6%)	5 (2.7%)	0.982	11 (2.7%)
Nº diuretics#@	0.93 ± 0.63	0.91 ± 0.81	0.82 ± 0.69	0.92 ± 0.76	0.817	0.89 ± 0.73

# Kolmogorov-Smirnov >0.05. Mean and standard deviation. Values are expressed as absolute numbers and percentage (in parentheses). @ Excluding MRAs and SGLT2i. Abbreviations: ACEI/ARB, angiotensin-converting enzyme inhibitors/angiotensin receptor blockers; ARNI, angiotensin receptor and neprilysin inhibitor; CA125, Carbohydrate antigen 125; MRA, mineralocorticoid receptor antagonist; SGLT2i, Sodium-glucose cotransporter 2 inhibitors.

**Table 4.** **Blood tests on admission**.

	FIB-4 <1.3	FIB-4 <1.3	FIB-4 ≥1.3	FIB-4 ≥1.3	*p*	All patients
	CA125 ≤50 U/mL	CA125 >50 U/mL	CA125 ≤50 U/mL	CA125 >50 U/mL		n: 402
	n: 43	n: 57	n: 117	n: 185		
Urea (mg/dL)	54.5 [38.0–87.5]	51.5 [37.5–72.8]	54.0 [39.0–92.0]	58.0 [40.0–84.0]	0.787	55 [39–83]
Creatinine (mg/dL)	1.1 [0.8–1.5]	1.2 [0.9–1.6]	1.3 [1.0–1.7]	1.3 [0.9–1.8]	0.227	1.2 [0.9–1.7]
Glomerular filtration rate (mL/min/1.73 m^2^)	61.0 [35.5–77.5]	48.0 [38.0–79.0]	47.0 [31.0–67.0]	51.0 [32.0–75.5]	0.182	49 [34–74]
Bilirubin (mg/dL)	0.5 [0.4–0.8]	0.9 [0.6–1.2]	0.7 [0.5–1.0]	0.9 [0.6–1.5]	<0.01	0.8 [0.5–1.2]
GOT/AST (U/L)	18.0 [12.0–22.0]	22.0 [18.0–25.0]	25.0 [20.0–35.0]	29.0 [22.0–40.0]	<0.01	25 [19–36]
GPT/ALT (U/L)	17.0 [13.0–31.0]	22.0 [16.0–38.5]	17.0 [12.0–24.0]	19.0 [13.0–32.0]	<0.01	19 [13–30]
usTnT (ng/L)	47.4 [32.9–103.4]	43.5 [26.4–64.2]	47.0 [23.0–98.4]	43.8 [29.4–97.0]	0.819	46 [27–93]
NT-proBNP (pg/mL)	3501 [2534–8058]	6673 [3755–14,153]	5739 [2416–10,394]	7299 [3474–14,363]	<0.01	6336 [2966–12,328]
Sodium (mEq/L)	140 [136–142]	138 [135–141]	140 [137–142]	138 [136–141]	0.01	139 [136–141]
Potassium (mEq/L)	4.3 [3.8–4.9]	4.2 [3.7–4.6]	4.3 [4.1–4.6]	4.2 [3.8–4.6]	0.114	4.2 [3.9–4.6]
Hemoglobin (g/dL)	12.7 [11.5–13.5]	12.2 [9.9–13.2]	12.5 [10.9–14.2]	12.3 [10.6–13.7]	0.423	12.3 [10.7–13.8]
Hematocrit (%)	40.1 [36.3–43.1]	39.0 [34.1–42.8]	39.3 [35.0–44.9]	39.1 [34.2–43.6]	0.742	39 [35–44]
Platelets (×10^3^/L)	293 [225–332]	287 [231–336]	186 [149–231]	192 [161–241]	<0.01	209 [166–262]
Uric acid (mg/dL)	7.6 [6.2–8.6]	7.7 [6.0–9.7]	7.7 [6.3–9.0]	7.8 [6.3–9.5]	0.900	7.7 [6.3–9.3]
TSAT (%)	18.0 [12.0–25.2]	16.5 [11.0–21.8]	20.0 [14.0–27.8]	18.0 [13.0–25.0]	0.148	19 [13–26]
Ferritin (ng/mL)	88 [41–212]	172 [81–388]	177 [85–339]	179 [85–387]	0.05	165 [77–345]
FIB-4	0.9 [0.8–1.1]	1.0 [0.8–1.1]	2.4 [1.9–3.5]	2.4 [1.9–3.7]	<0.01	2.1 [1.3–2.9]
CA125 (U/mL)	31 [20–43]	137 [73–224]	24 [15–33]	140 [78–261]	<0.01	63 [30–158]

Values are expressed as absolute numbers and percentage (in parentheses). Abbreviations: ALT (GPT), alanine aminotransferase; AST (GOT), aspartate aminotransferase; CA125, Carbohydrate antigen 125; TSAT, transferrin saturation; NT-proBNP, N-terminal pro-B-type natriuretic peptide; usTnT, ultrasensitive troponin.

### 3.3 Echocardiographic Assessment

Reduced left ventricular ejection fraction (LVEF) was observed in 62% of patients. Approximately 28% of patients had left ventricular hypertrophy and 30.3% had severe left atrial enlargement. Right ventricular (RV) function was preserved in most cases (65%), although RV dilation was present in 53%. A normal-sized IVC with inspiratory collapse >50% was seen in 24% and estimated pulmonary artery systolic pressure was elevated in 29%.

Echocardiographic comparisons showed that the group with both biomarkers elevated had a higher prevalence of significant mitral regurgitation and tricuspid regurgitation (*p* < 0.05). Inspiratory IVC collapse was less frequent in this group (*p* < 0.05) (Table 5).

**Table 5.** **Echocardiographic evaluation**.

		FIB-4 <1.3	FIB-4 <1.3	FIB-4 ≥1.3	FIB-4 ≥1.3	*p*	All patients
		CA125 ≤50 U/mL	CA125 >50 U/mL	CA125 ≤50 U/mL	CA125 >50 U/mL		n: 402
		n: 43	n: 57	n: 117	n: 185		
Preserved LVEF (≥50%)		16 (37.2%)	17 (29.8%)	34 (29.1%)	45 (24.3%)	0.359	112 (27.9%)
Dilated LV (LV-EDD >56 mm)		8 (18.6%)	14 (24.6%)	29 (24.8%)	43 (23.2%)	0.867	94 (23.4%)
LVH (>12 mm)		13 (30.2%)	19 (33.3%)	33 (28.2%)	47 (25.4%)	0.675	112 (27.8%)
Severe left atrial dilatation (≥50 mm)		11 (25.6%)	18 (31.6%)	32 (27.4%)	61 (33.0%)	0.658	122 (30.3%)
Significant valvulopathies*							
	AoR	2 (4.7%)	0 (0.0%)	4 (3.4%)	4 (2.2%)	0.165	10 (2.5%)
	AoS	5 (11.6%)	2 (3.5%)	16 (13.7%)	18 (9.7%)	0.215	41 (10.2%)
	MR	4 (9.3%)	5 (8.8%)	7 (6.0%)	32 (17.3%)	0.020	48 (11.9%)
	MS	2 (4.7%)	3 (5.3%)	0 (0.0%)	4 (2.2%)	0.178	9 (2.2%)
	TR	4 (9.3%)	9 (15.8%)	9 (7.7%)	37 (20.0%)	0.048	59 (15%)
RV function (TAPSE)&							
	Normal	29 (67.4%)	37 (64.9%)	88 (75.2%)	106 (57.3%)	0.141	260 (64.6%)
	Mild dysfunction	4 (9.3%)	4 (7.0%)	15 (12.8%)	26 (14.1%)		49 (12.2%)
	Moderate dysfunction	8 (18.6%)	9 (15.8%)	7 (6.0%)	31 (16.8%)		55 (13.7%)
	Severe dysfunction	2 (4.7%)	7 (12.3%)	7 (6.0%)	22 (11.8%)		38 (9.5%)
Dilated RV (Basal diameter >40 mm)		6 (14.0%)	12 (21.1%)	26 (22.2%)	52 (28.1%)	0.205	96 (23.9%)
Inferior vena cava (mm)#		19.0 [17.5–21.5]	19.0 [18.0–23.0]	19.0 [18.0–23.0]	19.0 [18.0–23.0]	0.5485	19 [18.0–23.0]
Vena cava collapse ≥50%		24 (55.8%)	23 (40.4%)	53 (45.3%)	56 (30.3%)	<0.01	156 (38.8%)
PH (PAsP ≥50 mmHg)		8 (18.6%)	17 (29.8%)	30 (25.6%)	61 (33.0%)	0.226	116 (28.9%)

*Moderate-severe + severe. Significant valvular disease accounted for 41.5% of all patients. # Kolmogorov-Smirnov <0.05. Median and interquartile range. Values are expressed as absolute numbers and percentage (in parentheses). & TAPSE Ranges: Normal: TAPSE ≥17 mm; Mild dysfunction: TAPSE 13–16 mm; Moderate dysfunction: TAPSE 10–12 mm; Severe dysfunction: TAPSE <10 mm. Abbreviations: AoS, Aortic stenosis; AoR, Aortic regurgitation; CA125, Carbohydrate antigen 125; LV-EDD, Left ventricular end-diastolic diameter; LVEF, Left ventricular ejection fraction; MS, Mitral stenosis; MR, Mitral regurgitation; PH, Pulmonary hypertension; LVH, left ventricular hypertrophy; PAsP, pulmonary artery systolic pressure; RV, right ventricle; TAPSE, tricuspid annular plane systolic excursion; TR, Tricuspid regurgitation.

### 3.4 Survival, Adverse Clinical Events, and Correlation Analysis

**Fig. 2.**
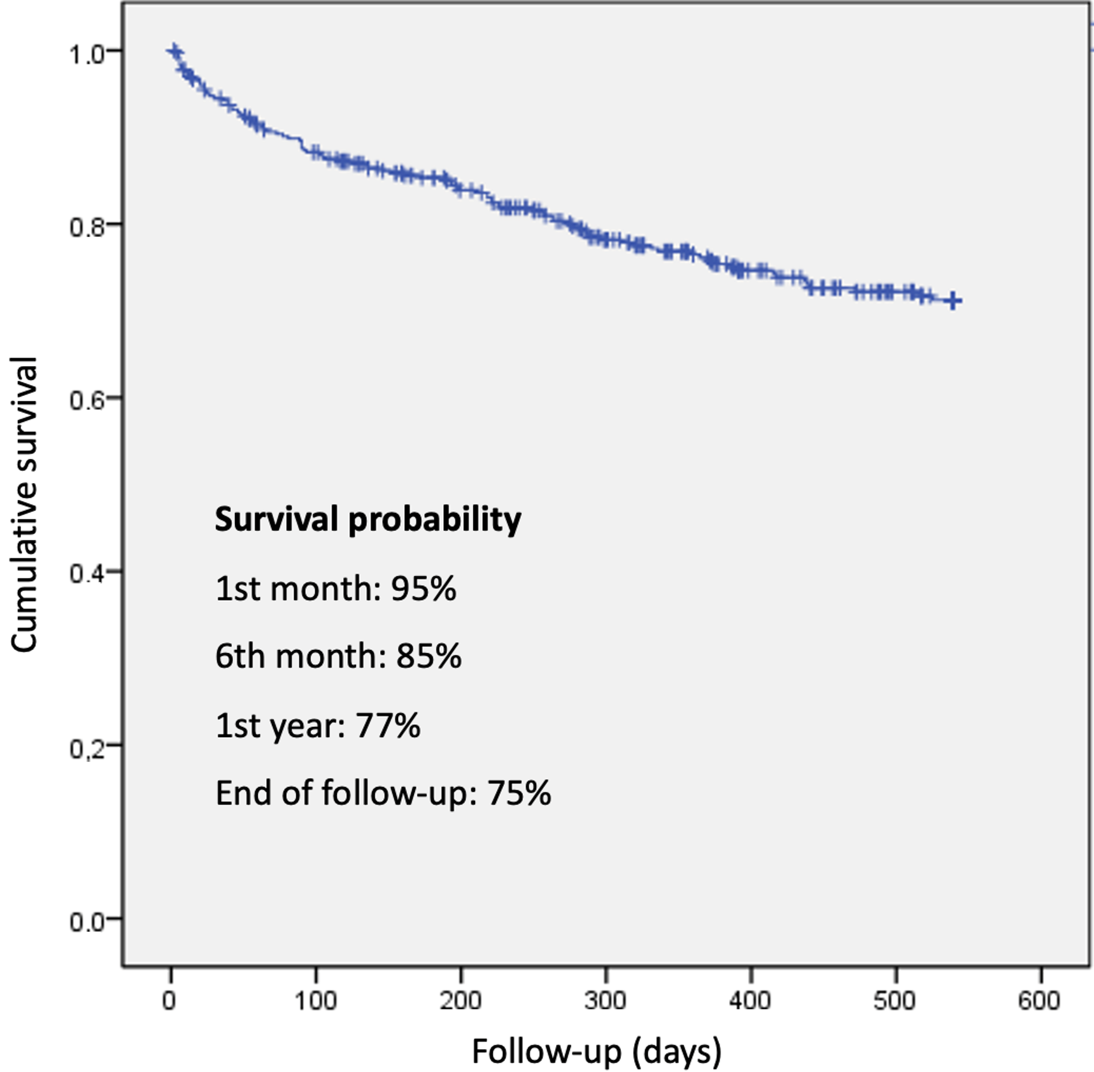
**Overall survival and survival by FIB-4 and CA125 groups**.

**Fig. 3.**
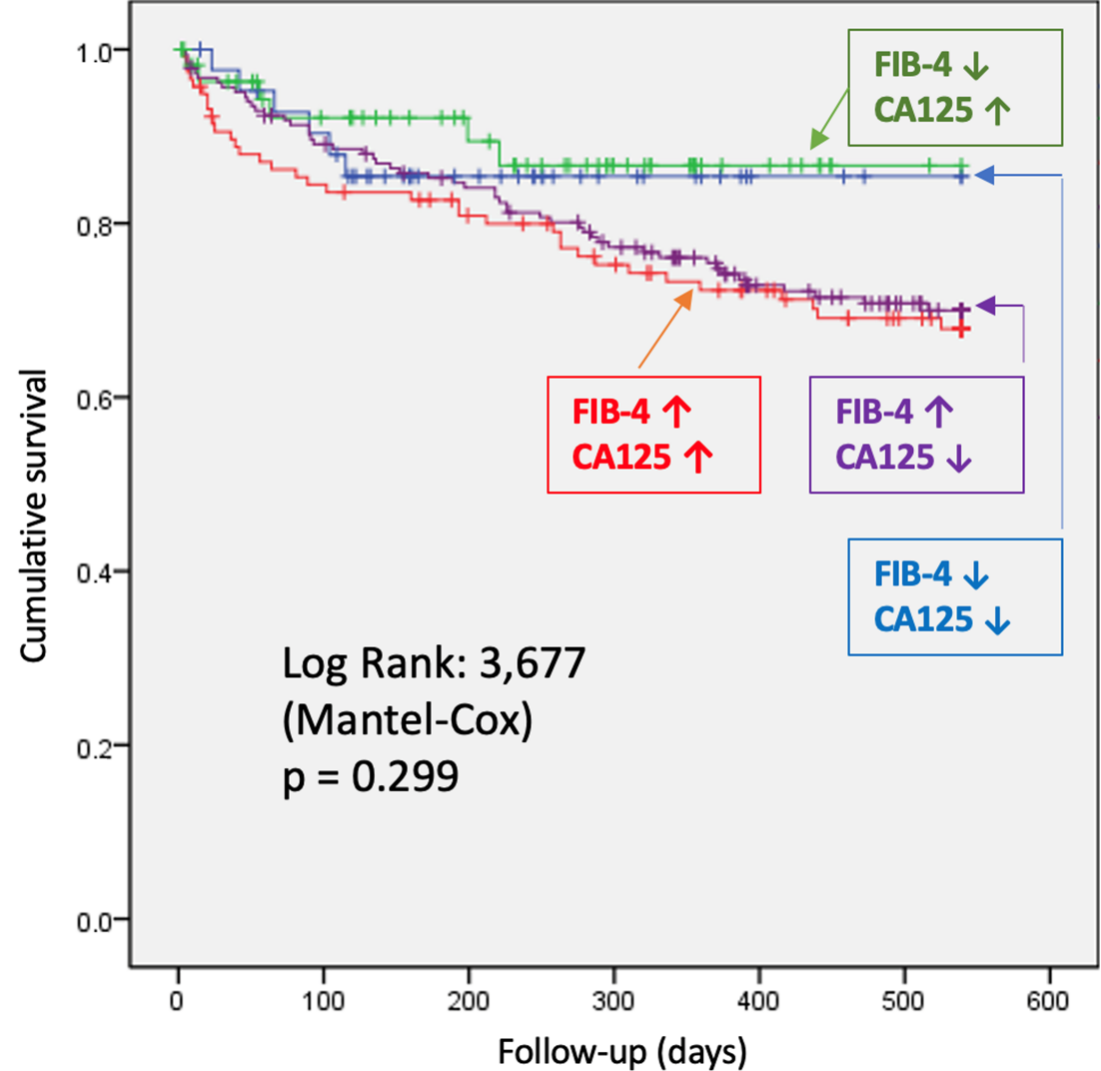
**Comparison of survival probability by study group**. Patients were categorized into four groups: blue (low FIB-4 [<1.3] and low CA125 [≤50 U/mL]), green (low FIB-4 and high CA125 [>50 U/mL]), purple (high FIB-4 [≥1.3] and low CA125), and red (high FIB-4 and high CA125). Censored observations are indicated by “+” symbols. Survival was compared among the four groups using the log-rank (Mantel–Cox) test (χ^2^ = 3.677, *p* = 0.299). Although curves begin to diverge after approximately 200–250 days, with the high FIB-4/high CA125 group showing the lowest cumulative survival, the differences did not reach statistical significance during the follow-up period. Comparison of survival probability by study group (Log-Rank test). Abbreviation: CA125, Carbohydrate Antigen 125.

**Fig. 4.**
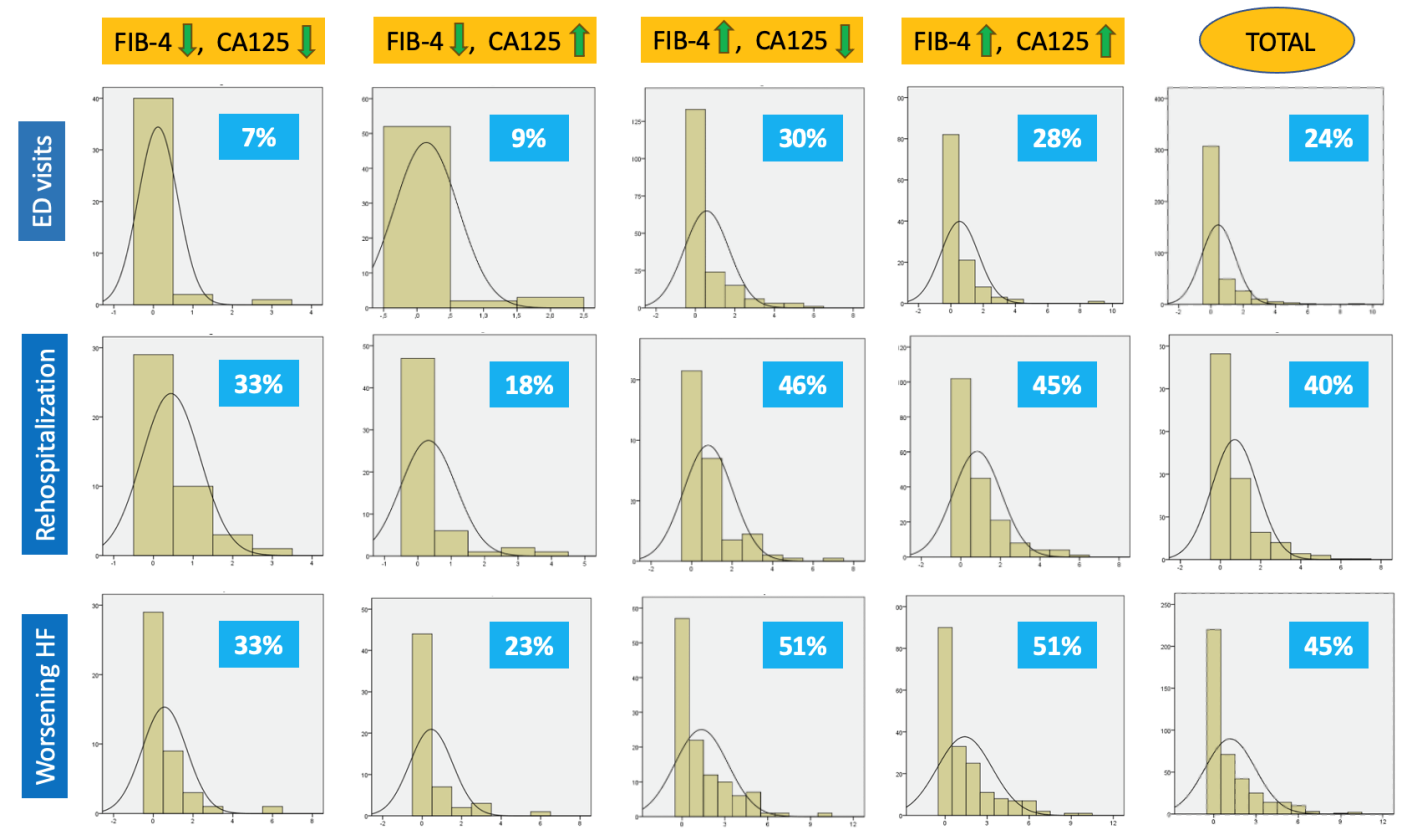
**Percentage of patients with the event by group**. Worsening HF is defined as the sum of emergency department visits requiring intravenous furosemide plus rehospitalization due to decompensated heart failure. Abbreviations: HF, Heart Failure; ED, Emergency Department; CA125, Carbohydrate Antigen 125.

**Fig. 5.**
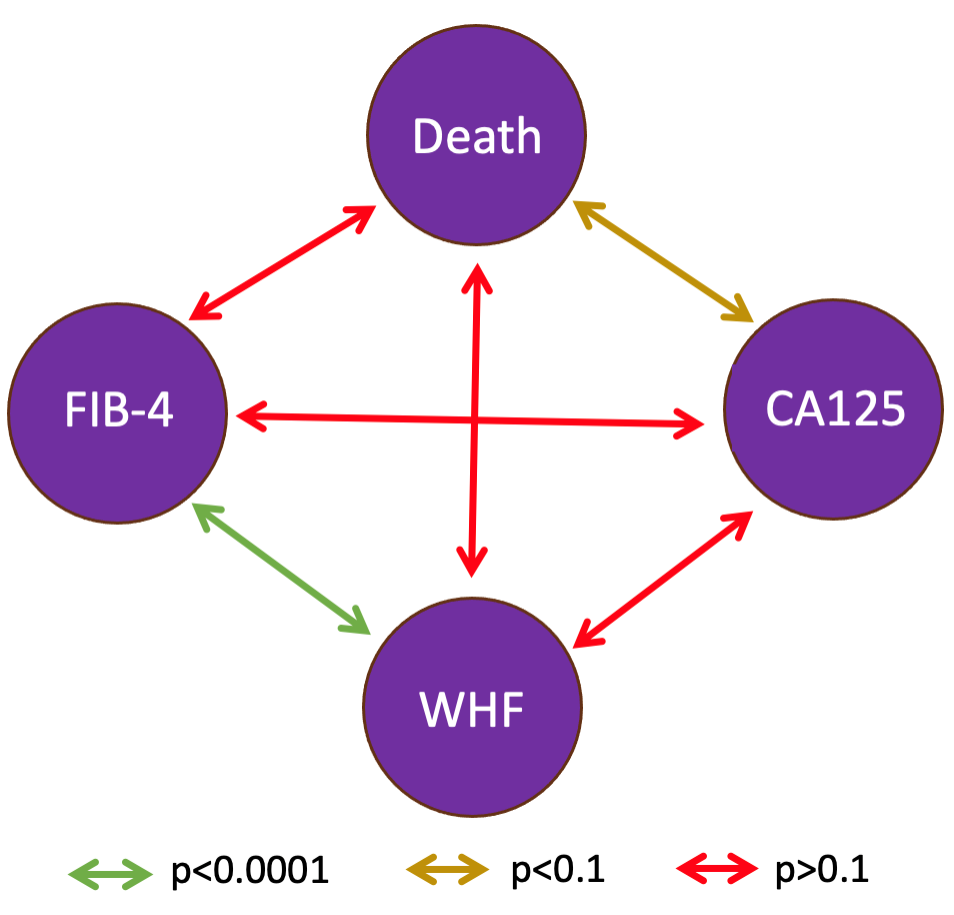
**Correlation between biomarkers and morbidity-mortality variables**. Abbreviations: CA125, Carbohydrate Antigen 125; WHF, Worsening Heart Failure.

Survival was 94.5% at 30 days and 76.1% at one year, with an overall mortality of 24.6%. No significant differences were found between groups (*p* = 0.29) (Table 6). Figs. 2,3 displays survival curves for the overall cohort and the four study groups. Although there were no significant differences in survival probability, curves begin to diverge after day 200–250, clustering according to FIB-4 values regardless of CA125 levels. Kaplan–Meier survival analyses stratified by FIB-4 and by CA125 separately are presented in **Supplementary Figs. 1,2**. The number of worsening events was high, with more than one episode per patient (mean 1.16), affecting nearly half the cohort, more often due to readmissions (40%) than emergency visits (24%). Worsening was more frequent in groups with elevated FIB-4, affecting more than 50% of these patients (*p* = 0.0001) (Table 6). Fig. 4 shows the number of events per patient and the percentage of affected patients in each group. No significant correlations were found between FIB-4 and CA125, or between either marker and mortality. A significant correlation was found only between FIB-4 and worsening (*p* < 0.0001). There was a trend toward a positive correlation between CA125 and mortality (*p* < 0.1) (Fig. 5).

**Table 6.** **Clinical outcomes by combined stratification of FIB-4 and CA125 levels**.

		FIB-4 <1.3	FIB-4 <1.3	FIB-4 ≥1.3	FIB-4 ≥1.3	*p*	All patients
		CA125 ≤50 U/mL	CA125 >50 U/mL	CA125 ≤50 U/mL	CA125 >50 U/mL		n: 402
		n: 43	n: 57	n: 117	n: 185		
Survival probability						0.299	
	1 month	0.976 (0.024)	0.963 (0.026)	0.905 (0.027)	0.957 (0.015)		0.945 (0.011)
	1 year	0.854 (0.055)	0.866 (0.052)	0.723 (0.043)	0.754 (0.032)		0.761 (0.023)
	End of follow-up	0.854 (0.055)	0.866 (0.052)	0.679 (0.045)	0.708 (0.035)		0.750 (0.023)
	No. of all-cause deaths	6 (14.0%)	6 (10.5%)	35 (29.9%)	52 (28.1%)		99 (24.6)
Emergency department visits						0.001	
	No. of visits	5	8	63	105		181
	No. of visits per patient	0.12	0.14	0.54	0.57		0.45
	No. of patients (%)	3 (7%)	5 (9%)	35 (30%)	52 (28%)		95 (24%)
	Minimum-maximum	0–2	0–3	0–9	0.6		0–9
Readmissions						0.004	
	No. of readmissions	19	18	95	153		285
	No. readmissions per patient	0.44	0.32	0.81	0.83		0.71
	No. of patients (%)	14 (33%)	10 (18%)	54 (46%)	83 (45%)		161 (40%)
	Minimum-maximum	0–3	0–4	0–7	0–6		0–7
Worsening HF episodes						0.0001	
	No. of episodes	24	26	158	258		466
	No. of episodes per patient	0.56	0.46	1.35	1.39		1.16
	No. of patients (%)	14 (33%)	13 (23%)	60 (51%)	95 (51%)		182 (45%)
	Minimum-maximum	0–6	0–6	0–10	0–10		0–10

Abbreviations: HF, Heart Failure; CA125, Carbohydrate antigen 125. Survival probabilities were estimated using the Kaplan–Meier method (standard error) and compared across groups with the log-rank test. The number of all-cause deaths is expressed as absolute values and percentages. For morbidity outcomes (emergency department visits, readmissions, and worsening HF episodes), the table shows absolute numbers, mean events per patient, and the proportion of affected patients; global comparisons were performed using the Chi-square test for categorical variables and Student’s *t* test or Mann–Whitney U test for continuous variables, as appropriate. *p* values correspond to overall between-group comparisons.

## 4. Discussion

HF is a highly prevalent condition that shortens life expectancy and significantly impairs quality of life [1]. Throughout its natural course, patients frequently experience clinical worsening, including hospital emergency visits due to congestion and readmissions for decompensation [14]. Some biomarkers, such as CA125 and FIB-4, have been associated with worse clinical trajectories, although their role in the context of HF is still being clarified [9].

The combined use of FIB-4 and CA125 may provide complementary prognostic information in AHF. CA125 is a recognized marker of congestion in AHF; however, in some cases, it may remain at low levels even during decompensation [15]. FIB-4, serves as an indirect marker of hepatic congestion, chronic liver fibrosis, and systemic inflammation [4]. Even mild FIB-4 elevations (≥1.3) have been associated with worse outcomes in HF, potentially identifying patients at risk before marked CA125 elevation occurs [16]. Although both markers are linked to systemic congestion, CA125 predominantly reflects interstitial and serosal fluid accumulation, whereas FIB-4 captures different pathophysiological domains [10, 17, 18, 19]. Their combination could therefore enhance risk stratification and help define distinct clinical phenotypes.

This shared pathophysiological link to congestion forms the basis for interpreting our findings. Prior studies have individually associated elevated CA125 or FIB-4 with adverse outcomes in HF, yet no previous work has explored their combined prognostic value in AHF. We recognize that these interpretations remain mechanistic hypotheses, as direct measurements of congestion were not performed, and further research is warranted to confirm these associations.

In this study, we observed that patients with both elevated FIB-4 and CA125 had poorer short- to medium-term outcomes, particularly in terms of clinical worsening (both HF readmissions and emergency visits for decompensation). However, no significant differences were found in survival within the two-year follow-up period.

The optimal thresholds for CA125 and FIB-4 in AHF are not well established. Most studies have adopted values from other clinical settings, such as CA125 >35 U/mL in oncology and FIB-4 thresholds of <1.3, 1.3–2.67, and >2.67 in hepatology [4, 20]. In AHF, only one study proposed a CA125 cut-off of <23 U/mL, but it was based on a retrospective cohort with heterogeneous measurement times [21]. We selected 50 U/mL to minimize variability and exclude minor, clinically irrelevant elevations, supported by our previous work linking CA125 >50 U/mL to worse outcomes [22]. For FIB-4, although various prognostic thresholds exist, Mohamed *et al*. [16] found that intermediate values (1.3–2.67) already predict higher mortality in reduced LVEF; thus, we adopted ≥1.3 to capture early signs of hepatic congestion.

In our cohort, patients with both biomarkers elevated more frequently presented systemic or mixed congestion. Prior studies report that CA125 has high sensitivity for systemic venous congestion, sometimes superior to NT-proBNP, whereas evidence for FIB-4 is more limited [23]. Our findings suggest an additive effect: in patients with CA125 >50 U/mL, a FIB-4 ≥1.3 nearly doubled the likelihood of systemic/mixed congestion (38.6% vs 59.5%; OR 2.33, 95% CI 1.27–4.29).

Consistent with prior AHF studies, our cohort had a high mean age (>70 years). Older patients often present with comorbidities, polypharmacy, and frailty, which can complicate management [24]. In this setting, combining FIB-4 and CA125 may add value for geriatric risk stratification, as congestion markers could be particularly informative for prognosis and treatment decisions.

Admission NT-proBNP levels, although less specific for systemic congestion than CA125, are well established as prognostic markers [25]. In our study, NT-proBNP was highest in the high FIB-4 + high CA125 group, supporting the hypothesis that these patients had greater congestion and worse outcomes. Conversely, those with both indices normal had the lowest NT-proBNP levels, consistent with a lower-risk profile.

Among echocardiographic findings, 28% of patients had preserved LVEF (≥50%), with no significant differences across groups. Literature shows CA125’s prognostic value is independent of LVEF phenotype, whereas evidence on FIB-4 is more heterogeneous [16, 26]. Tseng *et al*. [27] found that FIB-4 was only associated with adverse outcomes in patients with preserved or mildly reduced LVEF. This has been attributed to the higher prevalence of hepatic steatosis and a pro-inflammatory state in these phenotypes, which may enhance the impact of hepatic congestion [28].

Regarding the right ventricle (RV), both CA125 and FIB-4 have been associated with RV dysfunction and systemic congestion [29]. In our cohort, severe RV dysfunction was more frequent in the high-risk group (11.8% vs 4.7%) but not statistically significant. However, other indirect indicators of central venous hypertension, such as reduced IVC collapsibility and higher prevalence of significant tricuspid regurgitation (TR), were significantly more common in this group. Some authors suggest that TR and reduced IVC collapsibility may be more sensitive indicators of central venous hypertension than RV dysfunction as estimated by tricuspid annular plane systolic excursion (TAPSE) [23, 30, 31]. Thus, the selected FIB-4 cutoff (≥1.3) may have favored detection of subtle degrees of congestion, manifested earlier through signs like IVC dilation and TR rather than overt RV dysfunction. Overall, our findings reinforce the predictive value of the clinical-echocardiographic pattern of systemic congestion captured by CA125 and FIB-4, which appear to offer complementary information.

One-year survival was 77%, with no significant differences at two years. However, curves began diverging at around six months, and there was a trend toward a positive correlation between CA125 and mortality (*p* < 0.1). In literature, higher values of both markers have been associated with worse prognosis. FIB-4, for instance, was evaluated in a registry of 1854 AHF patients and higher values were associated with increased 5-year all-cause mortality (HR: 1.009, 95% CI: 1.010–1.015) [27]. Thresholds similar to ours showed that FIB-4 >1.3 correlates with a 5-year mortality of 36%, compared to 23% for values <1.3 (HR: 1.33, 95% CI: 1.16–1.52). This risk rises further with FIB-4 >2.67 (mortality: 46%, HR: 2.14, 95% CI: 1.67–2.74) [16]. A multivariate analysis indicated that the prognostic value lies in the FIB-4 score itself, rather than in the individual components [32]. Furthermore, a meta-analysis of 16 studies involving 8401 AHF patients found that elevated CA125 was associated with increased risk of all-cause mortality (HR: 1.44, 95% CI: 1.21–1.72; *p* < 0.001) [33]. Interestingly, the association between CA125 >50 U/mL and mortality only becomes statistically significant beyond two years of follow-up [22]. One advantage of FIB-4 compared to CA125 is its ability to be reassessed over short intervals, as CA125 may require up to 10 days to reflect changes after an intervention [34]. Maeda *et al*. [35] studied 877 hospitalized AHF patients and showed that a reduction in FIB-4 of less than 1% during admission doubled the risk of mortality or rehospitalization, compared to reductions greater than 27% (HR: 2.16, 95% CI: 1.41–3.32; *p* < 0.001), regardless of baseline values.

Therefore, the absence of survival differences in our cohort may be influenced by the relatively short follow-up period, lack of post-discharge FIB-4 reassessment, and the low cutoff point (1.3 instead of 2.67).

In this study, the combination of CA125 and FIB-4 showed statistically significant differences regarding worsening events, identifying a very high-risk subgroup and a low-risk subgroup (51% vs 33% at two years; *p* < 0.001). For example, extrapolating results from the STRONG-HF trial to a two-year horizon assuming a linear progression, the rehospitalization rate in the best-prognosis arm (intensified therapy) would be comparable to that of our low-risk group (38% vs 33%) [36]. These findings suggest that identifying patients with low CA125 and FIB-4 could enable more accurate risk stratification, and, in resource-limited settings, help prioritize interventions for those at greatest risk.

From our perspective, both CA125, and especially FIB-4, are still under investigation, and further research is needed to define their real clinical utility, both individually and in combination.

This study has several limitations, mainly related to its retrospective design. Selection bias was minimized by the small baseline differences between groups, recall bias by prospective data entry at discharge by experienced cardiologists, and missing data bias by consecutive patient inclusion with predefined exclusion criteria. All relevant variables were included to reduce confounding. Another limitation is the imbalance in subgroup sizes, which may have reduced statistical power, particularly in survival analyses; however, this reflects the real-world distribution of biomarker profiles in AHF, and non-significant results in small subgroups were interpreted with caution. In addition, recurrent-event models were not applied; survival was analyzed as time to first event and other outcomes were reported descriptively, which may not fully address intra-patient clustering but was considered appropriate for this exploratory design. Furthermore, we did not perform multivariable Cox regression as neither FIB-4 nor CA-125 showed a significant association with the primary endpoint in univariate analysis. Including non-significant variables could lead to an overfitted and non-informative model. Finally, although few studies have evaluated the prognostic role of FIB-4 and none its relationship with CA125, we believe our findings are of scientific interest and provide a basis for future prospective research in larger cohorts with longer follow-up.

## 5. Conclusion

FIB-4 is a simple and useful indicator that may have prognostic implications in patients with decompensated AHF, helping to predict post-discharge outcomes. Values above 1.3, when combined with serum CA125 levels greater than 50 U/mL, identify a subgroup of patients who, although they do not show increased short- to mid-term (2-year) mortality, do present a significantly higher probability of clinical worsening, including HF readmissions and emergency department visits due to decompensation. Longer prospective studies with larger patient cohorts are needed to confirm the results of this analysis.

## Availability of Data and Materials

The datasets generated for this study contain sensitive clinical information and cannot be made publicly available due to patient confidentiality and institutional restrictions. Data may be made available from the corresponding author upon reasonable request, subject to ethics committee approval.

## Supplementary Material

Supplementary material associated with this article can be found, in the online version, at https://doi.org/10.31083/RCM42797.
