# Supplementary material for: Combination of the Fibrosis 4 Index and Carbohydrate Antigen 125 to Predict Morbidity and Mortality in Acute Heart Failure
Source: Rev Cardiovasc Med. 2025 Dec 22;26(12):42797. doi: 10.31083/RCM42797 (PMC12780997; doi:10.31083/RCM42797)
Supplement: Supplementary file 1 [file 2153-8174-26-12-42797-s1.pdf]

Figure S1

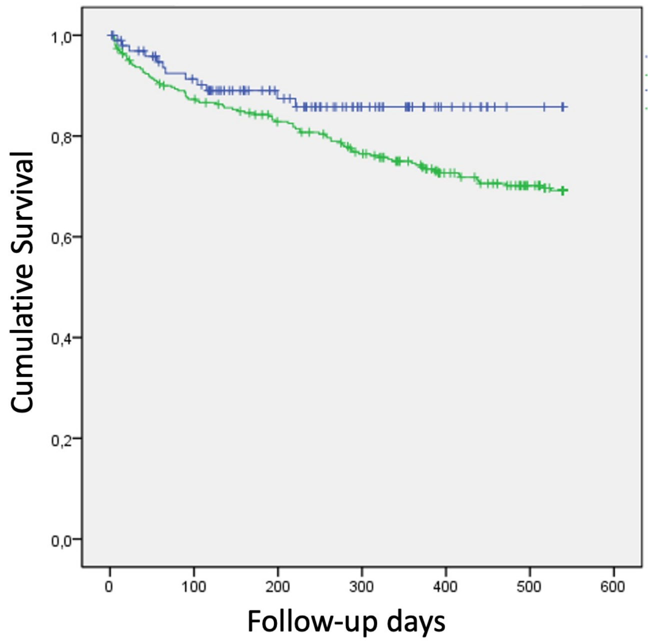

Value 1 (green) represents FIB-4 > 1.3, and value 0 (blue) represents FIB-4 < 1.3

Kaplan–Meier survival analysis showed a trend toward reduced survival in patients with elevated FIB-4 compared with those with lower values (28.8% vs 12.0% events; log-rank $\chi^2=3.25$, $p=0.071$). Although not statistically significant, the consistent separation of the curves suggests a clinically relevant association warranting confirmation in larger cohorts.

Figure S2

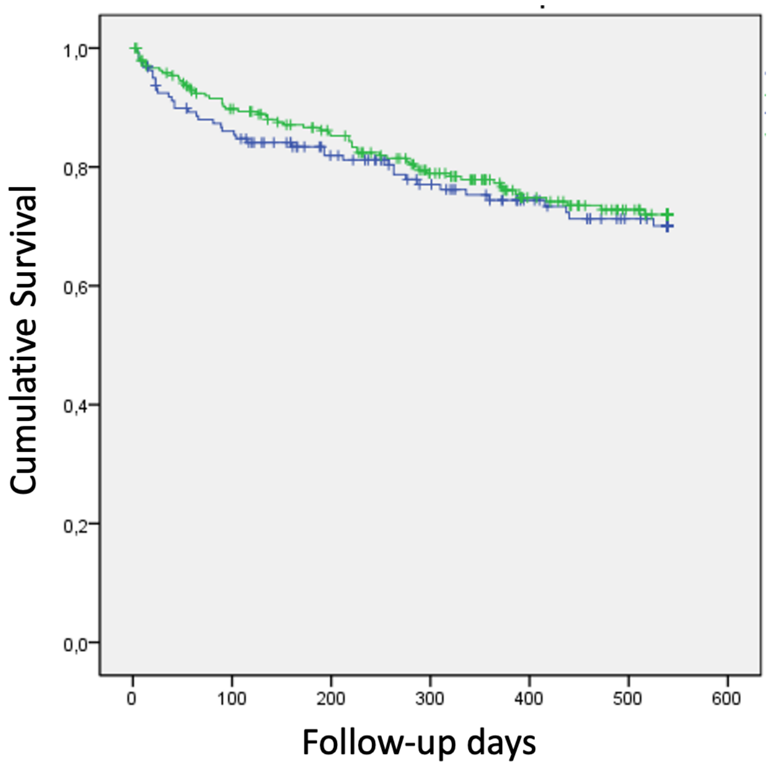

Value 1 (green) represents CA125 > 50, and value 0 (blue) represents CA125 < 50. Kaplan–Meier survival curves according to CA125 levels (≤50 vs. >50 U/mL). No significant difference in survival was observed between groups (log-rank p=0.572)

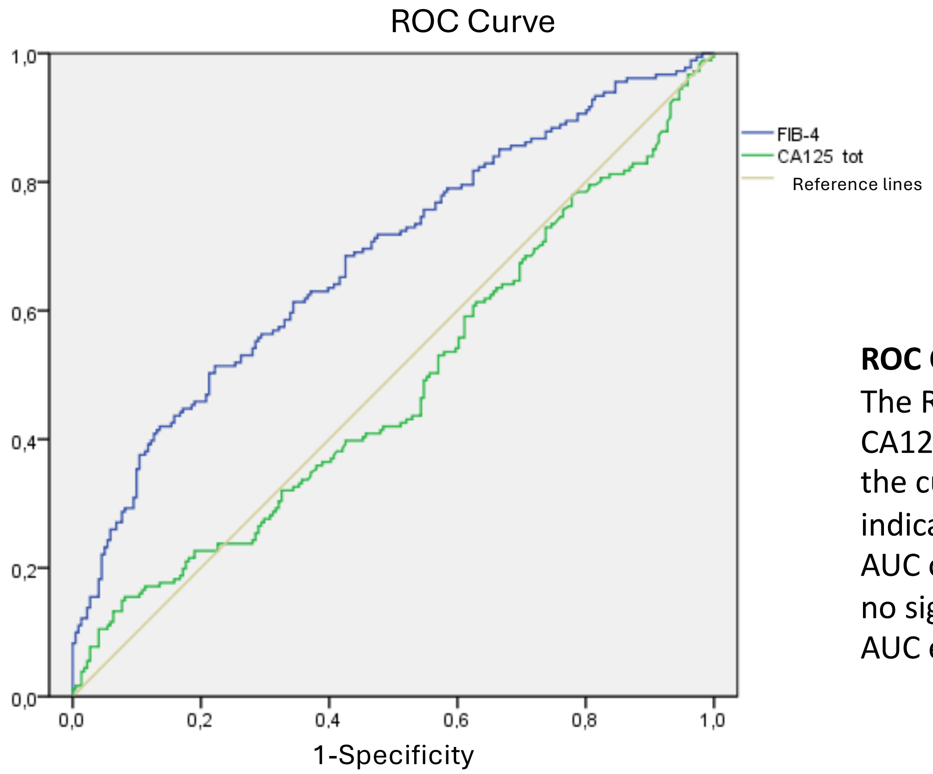

ROC Curve Analysis for FIB-4 and CA125

The ROC curve shows the discriminatory performance of FIB-4 and CA125 for the outcome of interest. FIB-4 demonstrated an area under the curve (AUC) of 0.682 (SE = 0.027, 95% CI: 0.630–0.735, $p < 0.001$), indicating moderate predictive ability. In contrast, CA125_tot had an AUC of 0.482 (SE = 0.029, 95% CI: 0.425–0.540, $p = 0.538$), suggesting no significant predictive value. The null hypothesis was that the true AUC equals 0.5.
